# Lightning Current Measurement Form and Arrangement Scheme of Transmission Line Based on Point-Type Optical Current Transducer

**DOI:** 10.3390/s23177467

**Published:** 2023-08-28

**Authors:** Jinming Ge, Yibo Yin, Wei Wang

**Affiliations:** School of Electrical Engineering, Northeast Electric Power University, Jilin 132012, China; gejinming@neepu.edu.cn (J.G.); 2202200124@neepu.edu.cn (Y.Y.)

**Keywords:** point-type optical current transducer, lightning measurement, measurement structure, transmission line

## Abstract

Lightning strikes are the main cause of transmission line faults, and the accurate lightning current number is an important basis to guide scientific lightning protection. The use of sensors with excellent sensing performance to carry out lightning current monitoring on transmission lines is beneficial to the accumulation of key parameters of original lightning strikes, so it is necessary to study the lightning current measurement structure of transmission lines. In this paper, an optical current-sensing unit is used to monitor the lightning current on transmission lines. A measuring structure that can monitor key parameters of the lightning current under different types of lightning strikes is proposed. First, establish the lightning current return channel model and the equivalent model of the tower, study the influence of the transmission tower on the current in the lightning channel, and analyze the direct measurement position of the lightning current on the tower; establish the multi-wave impedance model of the tower, and build a multi-base tower. The simulation model of the transmission system analyzes the transmission characteristics of the lightning current on the transmission line and the lightning protection line in the case of different types of faults; from the perspective of the measurement of key parameters of the lightning current, the lightning current measurement structure of the transmission system is constructed to analyze different lightning strikes. The measurement effect of each monitoring position in the case of a lightning strike and the waveform characteristics of the fault current in the case of insulator flashover are analyzed.

## 1. Introduction

With the continuous adjustment of energy equipment and the continuous development of transmission technology, the scope of the power supply is becoming more and more extensive, and transmission lines are becoming longer and longer [1]. Due to the fact that high-voltage transmission lines and most low-voltage transmission lines are currently installed at high altitudes, they are easily affected by lightning strikes, leading to lightning accidents [2]. Power system operation experience shows that lightning strikes have become the main cause of tripping high-voltage transmission lines, which has seriously affected the safety and reliable operation of the power grid [3]. At present, the lightning protection design of transmission lines at home and abroad is mainly carried out through simulation experiments, theoretical analysis, and accident research, lacking real lightning parameters. In the study of the lightning protection design of transmission lines, obtaining true and accurate lightning parameters is the basis of the lightning protection design of transmission lines [4,5]. Therefore, it is very urgent and necessary to use the lightning current sensor suitable for the transmission system to study the measurement form of the sensor on the transmission line and realize the measurement of the key lightning current parameters [6,7,8,9].

Since 1962, ref. [10] installed magnetic steel rods on the top lightning rod, the lightning wire and coupling ground wire on both sides of the tower head, the lightning protection cable on the four sides of the tower head, and a main angle steel of the tower. They have monitored the Zhejiang Xinhang line for decades. China’s relevant industry standards are also based on a large number of measured lightning data. In [11], magnetic card measuring devices are installed at the end and foot of the insulator string tower to measure the lightning current parameters (lightning current amplitude, rise rate) and record the lightning parameter characteristics flowing through the corresponding position. In [12], the differential ring is installed near the lightning protection lines and transmission lines, and a non-contact lightning current measurement method for transmission lines is proposed. But a magnetic steel rod, a magnetic card tape, and other devices can only measure the peak value, rise rate, and other key parameters of the lightning current. The non-contact measurement of the lightning current is realized by measuring the magnetic field of the lightning current. This shows that it is necessary to combine the lightning current distribution of different lightning strike types to study the measurement form [13].

From the above research, the arrangement of lightning current measurement sensors can be summarized as follows:

(1) Direct measurement to realize the accurate measurement of the full waveform of the direct lightning current. In [14], it mainly focuses on the situation of lightning strikes on towers. Current sensors and lead lightning conductors are installed on the tower top to measure the lightning current of towers at different altitudes on a ridge, and the data are collected through remote control. In [15], Rogowski coils are installed on the four tower feet of the tower, and they are connected by special modules to form a detection system, and the lightning strike data are collected by current sensors and voltage sensors. In [16], a current and field measurement system with broadband and high resolution was used to measure the lightning current and related electromagnetic fields of the CN tower.
(2) Long-distance measurement, based on the propagation characteristics of the lightning signal in the propagation medium, by measuring the lightning current propagating to the monitoring position, and then obtaining key parameters, such as the amplitude and polarity of the lightning current in a large range. The lightning measurement system proposed in [17] installs sensors on every 20–30 km of wire along the transmission line to accumulate lightning data across that distance. In [18], the current waveform was measured by installing Rogowski coils on the arrester.
(3) Fault measurement: measure the lightning current when a lightning fault occurs, obtain the key parameters of the lightning current that causes the fault, and infer the fault type through the measurement information. In [19], a Rogowski coil was installed on the suspension insulator string to detect the V-t characteristics of the suspension insulator string for the fault caused by the lightning strike in a DC traction power supply system. The lightning measurement system proposed in [20] nests a Rogowski current sensor on the side hardware of the insulator string tower of the transmission line tower to measure the flashover current during the lightning strike and then calculates the lightning current amplitude.

The above monitoring systems, with different measurement forms, have achieved certain results. However, the installation position of the measuring device is mostly determined based on practical experience, lacking a theoretical analysis. Optical current-sensing technology, as a new type of current-sensing technology, is currently widely used for current monitoring in intelligent substations. It has advantages, such as high insulation strength, a large dynamic range, a wide frequency band, strong anti-interference ability, no magnetic saturation, ferromagnetic resonance, a small size, a light weight, and a low cost. In principle, it is very suitable for measuring lightning currents [21].

In this study, the application of optical current-sensing technology, which can meet the waveform measurement requirements of a lightning current, is taken as the research background, and the measurement of lightning current parameters at the installation position of sensors on transmission lines is analyzed. An equivalent model of lightning strikes on transmission towers has been established to study the impact of transmission towers on the current in lightning channels. Then, starting from the perspective of measuring key parameters of the lightning current, a measurement structure for the lightning current in the transmission system is constructed, and the measurement effects of different monitoring positions under different lightning strikes are analyzed. The waveform characteristics of the fault current under insulator flashover are analyzed. This article mainly studies lightning current monitoring, aiming at the waveform and characteristics of the lightning current, reflecting the accumulation of data, which can lay a foundation for differentiated lightning protection designs in the future.

## 2. Principle of the Optical Current Transformer and Feasibility of Measuring the Lightning Current

### 2.1. Characteristics of the Lightning Current Waveform

#### 2.1.1. Typical Lightning Current Waveform

**Figure 1:**
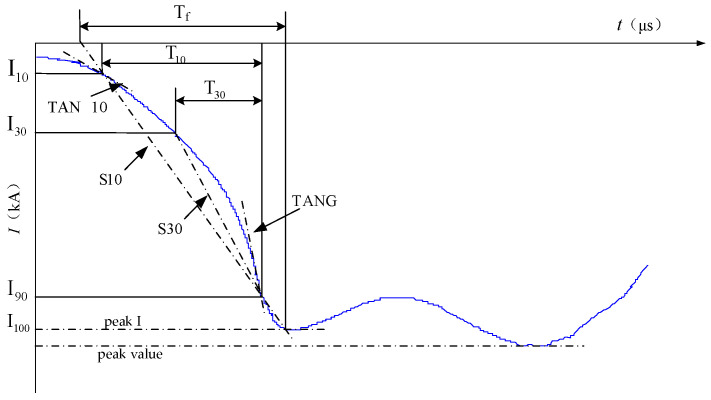
Waveform of typical first return stroke current.

The waveform of a lightning current can not only provide important parameters that need statistical application in lightning protection design, such as the amplitude, rise rate, and wavelength of the lightning current, but also provide a research basis for the study of the physical process of lightning formation. A lightning current can be divided into the positive and the negative, in which negative lightning accounts for more than 90% of the cloud–ground lightning strikes. The lack of monitoring data of positive lightning makes it impossible to extract the general characteristics of positive lightning and summarize the typical waveforms of positive lightning. The first return stroke waveform of negative lightning adopted by the Institute of Electrical and Electronics Engineers (IEEE) is shown in Figure 1.

The peak I in the figure is the first peak of the lightning current amplitude (kA); the peak value is the maximum value (kA) of the lightning current amplitude; *T_f_* is the wave head time (μs); *T_t_* is the duration of the lightning current from zero to half peak, which is the wavelength time (μs); *T*_10_ is the time when the value of peak I increases from 10% to 90% (μs); *T*_30_ is the time when the value of peak I increases from 30% to 90% (μs); S_10_ represents the average rate of increase in the peak I value from 10% to 90% (kA/μs); S_30_ represents the average rate of increase in the peak I value from 30% to 90% (kA/μs); and TANG is the maximum rise rate of the current waveform (kA/μs). It can be noted that before the current reaches the peak value, there is an obvious concave surface at the maximum gradient of the waveform. Some scholars believe that the concave surface is formed because the downward negative leader is not connected with the connecting leader at the initial stage of lightning development, and the discharge phenomenon is weak. Therefore, the lightning current waveform has a low gradient. When the two are connected, the instantaneous strong discharge phenomenon occurs, and the maximum gradient of the waveform generally occurs at this time. When the subsequent return lightning discharges along the channel of the first return lightning, its waveform will not appear concave [22].

The above lightning parameters can be divided into three categories: amplitude (peak I, peak value), duration (*T_f_*, *T_t_*, *T*_10_, *T*_30_) and rise rate (S_10_, S_30_, TANG). Different types of lightning current parameters will cause different types of faults: (1) the amplitude of the lightning current has a great influence on the power system. A small amplitude may lead to the transmission line strike accident, while a large amplitude may lead to lightning-induced overvoltage and lightning wave intrusion in substation. (2) Statistics show that 50% of lightning strike accidents in power systems are related to the lightning current rise rate, which can cause hidden dangers and accidents, such as an inductance effect of the lightning protection grounding body, electromagnetic induction overvoltage of secondary equipment, and transmission line overvoltage caused by back-flashover. (3) In the lightning impact experiment of electrical equipment, the characteristics of lightning impact volts per second of electrical equipment show that the length of the wave head time is closely related to the insulation strength of the electrical equipment, and the wavelength duration T_t_ mainly affects the power system in the form of a thermal effect. The degree of lightning discharge heat can be evaluated by obtaining the integral of I^2^dt. Therefore, the above parameters of the lightning wave are very important in the design of line lightning protection, grounding device design, arrester design, and rotary motor, transformer, and other electrical equipment insulation design.

#### 2.1.2. Expression Method of the Lightning Current Waveform

**Figure 2:**
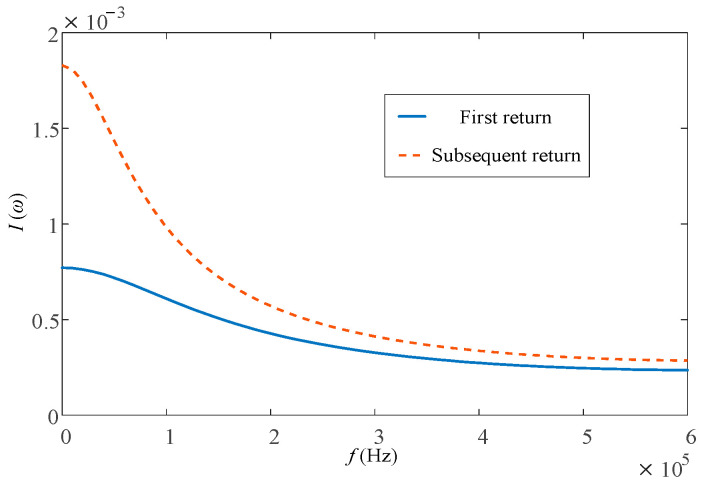
Lightning current amplitude spectrum.

The Heidler function model is used to simulate the lightning current source. Heidler function models with waves of 2.6/50 μs and 0.25/100 μs were used to simulate the first and subsequent return lightning currents, and Fourier transform was performed on them. The spectrum curve is shown in Figure 2. As can be seen from Figure 2, the frequency band of lightning flow is relatively wide, but the amplitude of lightning flow decreases with the increase in frequency, and the amplitude is mainly concentrated in the low-frequency part.

### 2.2. Optical Current Transducer

#### 2.2.1. Operation Principle of the Optical Current Transducer

**Figure 3:**
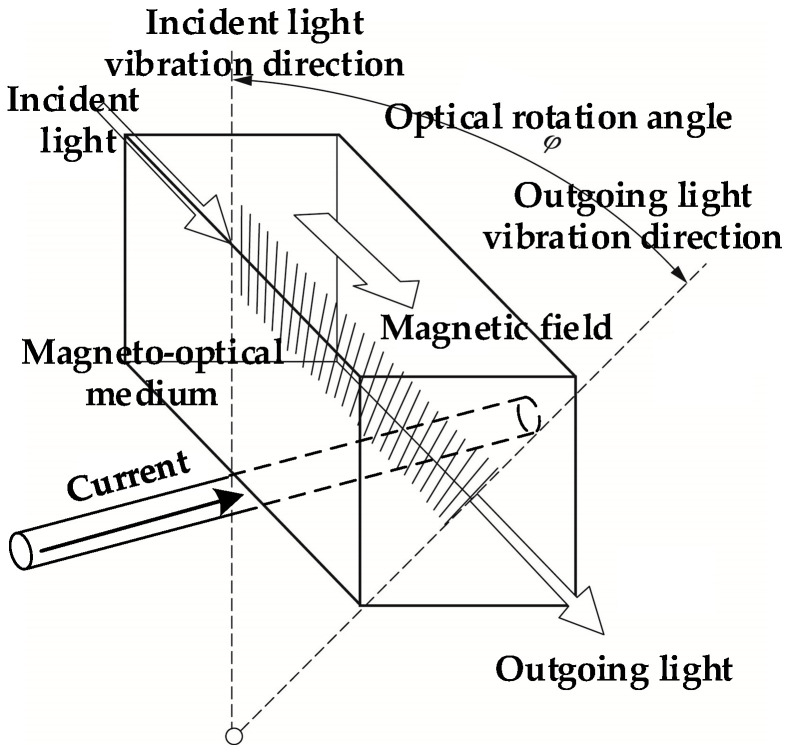
Schematic diagram of Faraday magneto-optical effect.

The sensing principle of the optical current transducer can be described as follows [23]. When a beam of linearly polarized light passes through a magneto-optical medium in a magnetic field, under the action of the magnetic field component along the propagation direction of the linearly polarized light, the polarization plane of the linearly polarized light will deflect. The diagram of the Faraday magneto-optic effect is shown in Figure 3.

When deflection occurs, the deflection angle is the Faraday rotation angle *φ*, and the *φ* angle can be expressed as follows:

(1) $$\varphi =\frac{V}{\mu }\int B\cdot dL$$

where *V* is the Verdet constant of the magneto-optical medium, *μ* is magnetic permeability, **B** is magnetic induction intensity vector, and **L** is the length vector of the propagation path of linearly polarized light along the direction of the magnetic field.

When the optical path is a closed path, such as an all-fiber circuit transducer or a closed optical glass transducer, the sensing principle can be expressed as follows:

(2) φ=NVI

where *N* is the number of turns of the optical path around the conductor to be tested, and *I* is the current to be measured.

#### 2.2.2. Point-Type Optical Current Transducer

The Faraday Magneto-optic effect reveals the interaction between linearly polarized light and the magnetic field, which can be further used to measure the current. An optical current transducer has the advantages of a high dielectric strength, large dynamic range, no bandwidth limitation, strong anti-interference ability, no magnetic saturation and ferromagnetic resonance, a small size, a light weight, and a low cost. From the perspective of the measurement principle, it is very suitable for the measurement of a lightning current.

According to the measurement principle, a point-type optical current transducer (POCT) composed of strip-shaped magneto-optical materials can be designed as a sensing unit, which is arranged at a certain position around the conductor for current measurements. Compared with closed magneto-optical glass, the sensing unit of POCT uses fewer optical components and has a simple overall structure, which greatly improves the overall stability of the sensing unit. In addition, the POCT sensing unit can be suitable for different insulation requirements of the tested conductor and can be flexibly installed in different measurement environments, suitable for the research of lightning current measurements on transmission lines.

The experimental impulse current waveform refers to the national standard [24], and the waveform generated by the impulse current generator is 8/20 μs. And the Pearson sensing unit is used as a standard current transformer to measure the original waveform of the impulse current source.

**Figure 4:**
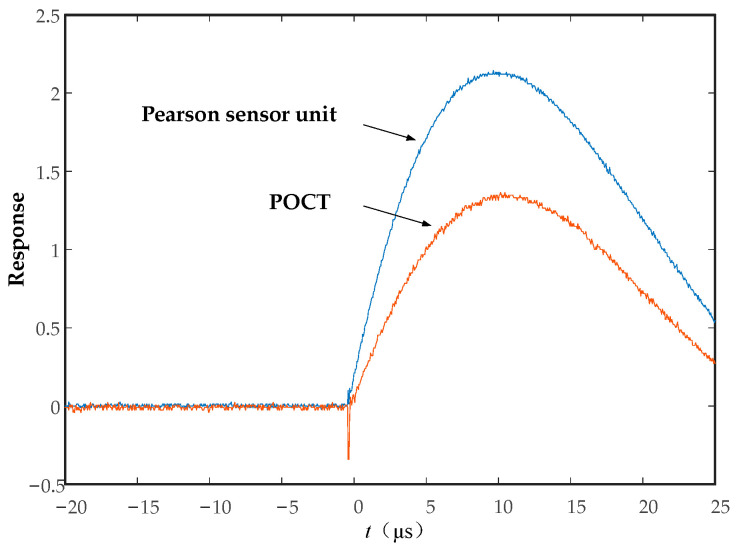
Impulse current waveform.

The impulse current waveform obtained from the Pearson sensing head unit and POCT is shown in Figure 4.

As can be seen from Figure 4, the POCT can realize the measurement of key parameters, such as the amplitude, rise rate, duration, and waveform of the lightning current, and can provide hardware support for the measurement of the lightning current on transmission lines.

## 3. Measurement Form Based on POCT

### 3.1. Direct Measurement of Lightning Current on Tower Based on the POCT

In the direct measurement of the lightning current, due to the direct measurement method and less interference, the measurement results can not only be applied to the accumulation of local lightning strike data, but also be applicable to the application of lightning current engineering models.

#### 3.1.1. Equivalent Model of Lightning Strike Tower

Scholars have conducted research on the formation process of lightning currents after lightning strikes tall buildings [25]. These studies assumed that when a lightning strike occurs, the first return current strike propagates upward from the ground, and the current division in the lightning channel can be expressed as follows:

(3) i(z,t)=P(z)i(0,t−z/v)

where *z* is the height from the ground in the lightning channel, *i*(0,*t*) is the channel current at the ground, *v* is the speed of the return stroke, and *P*(*z*) is the current attenuation coefficient.

According to the MTLE transmission line current model [26], the return-stroke current decreases exponentially as the height increases, and Formula (3) can be expressed as follows:

(4) $$i(z,t)=e^{-z/\lambda }i(0,t-z/v)$$

where *λ* is the attenuation coefficient.

In the engineering model analysis of the lightning current return stroke, it can be considered that there are infinitely many current elements distributed in the lightning channel [27]. When the return-stroke front reaches a certain height of the channel, the charge stored in the pilot corona sheath is absorbed into the return-stroke wave head, and the current element corresponding to the charge is instantaneously activated and travels downwards at the speed of light. The current element at height *z* can be expressed as follows:

(5) $$\begin{array}{ll} di_{s}(z^{\prime },t)=0 & t<z^{\prime }/v\\ di_{s}(z^{\prime },t)=f(t-z^{\prime }/v)e^{-z^{\prime }/v}dz & t\geq z^{\prime }/v \end{array}$$

where *f(t)* is the function corresponding to the return-stroke model and current element.

At this time, the current at height *z* in the lightning channel can be expressed as follows:

(6) $$i(z,t)=\int_{z}^{H}di_{s}(z^{\prime },t-\frac{z^{\prime }-z}{c})=\int_{z}^{H}e^{-z/\lambda }f(t-\frac{z^{\prime }}{v}-\frac{z^{\prime }-z}{c})dz^{\prime }$$

where *c* is light speed, *H* is the channel current at the ground, *v* is the effective length of lightning channel, and *H* = (*t* + *z*/*c*)/(1/*v* + 1/*c*).

The current at the bottom of the channel can be obtained as follows:

(7) $$i(0,t)=\int_{0}^{H}e^{-z/\lambda }f(t-\frac{z^{\prime }}{v}-\frac{z^{\prime }}{c})dz^{\prime }$$

The above analysis results are obtained under the assumption that the lightning channel starts from the ground, and the lightning channel matches the wave impedance of the ground. However, when a lightning strike actually occurs, it is necessary to study the distribution of the current when the lightning strikes the building and there is a current traveling wave refraction at each contact point. In addition, the inconsistency of the building structure of the ground mine body itself will affect the monitoring results [28]. To simplify the analysis, this part takes the wine-glass-shaped straight tower as an example, and the transmission tower is equivalent to a non-destructive single wave impedance line. Due to the time-varying characteristics of the lightning current in the soil spark discharge area around the grounding body of the tower, the impact grounding resistance of the tower is also time-varying. However, due to the significant difference between the equivalent impedance of the tower and the grounding impedance, its time-varying characteristics basically do not affect the lightning current reflection at the contact point. In the analysis, it can be considered that the grounding impedance of the tower is a constant value.

**Figure 5:**
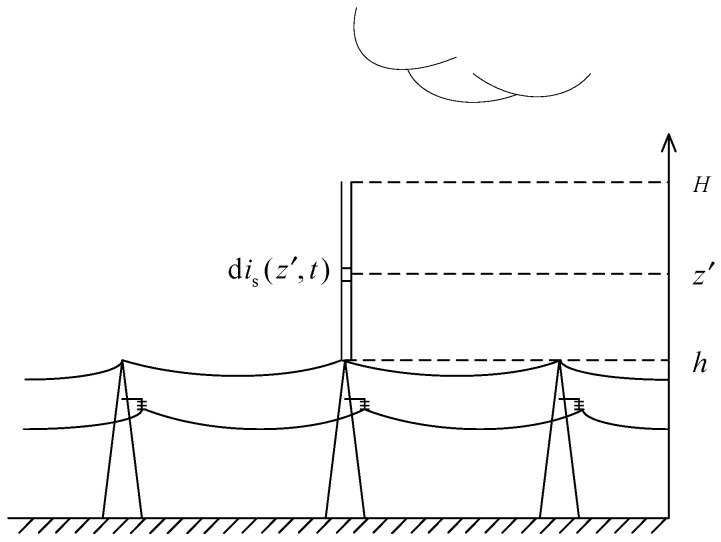
Schematic diagram of a lightning strike on a tower.

The schematic diagram of when lightning strikes the transmission tower is shown in Figure 5. The lengths of the lightning protection lines between the two gears are *l*_gl_, and *h* is the height of the tower. It is considered that the equivalent wave impedance of the lightning channel is *Z*_ch_, the vertical part of the transmission tower is *Z*_t_, the equivalent impedance of the grounding system is *Z*_g_, and the equivalent impedance of the transmission line and lightning protection line are *Z*_gl_ and *Z*_tl_, respectively.

**Figure 6:**
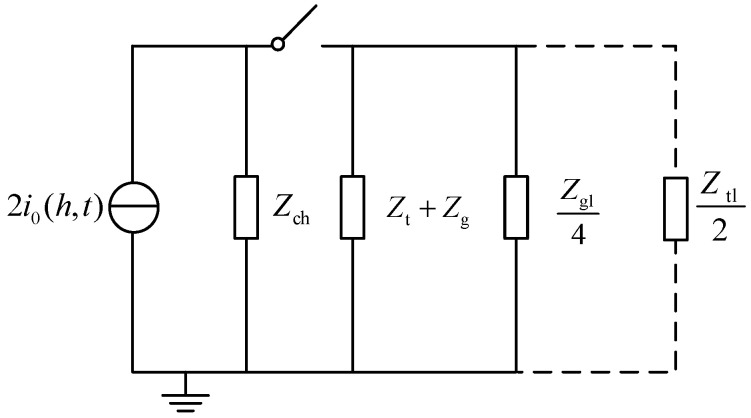
Equivalent circuit diagram of the lightning strike transmission tower.

The equivalent circuit of lightning striking a tower is shown in Figure 6. *I*_0_ (*h*, *t*) is the current, assuming that the equivalent impedances of different conductors match each other, that is, the contact point does not reflect, which is called the ideal current. When the switch is closed, lightning strikes the tower. Part of the lightning current enters the ground along the base tower, and part of the lightning current is transmitted to the adjacent tower through the lightning wire. After flashover of the insulator string caused by the lightning stroke, the shunt effect of the broken phase conductor shall also be considered, as shown in the dotted line in Figure 6.

The lightning traveling wave passing through any conductor *i* connected to the tower top, the reflection coefficient reaching the struck tower top can be expressed as follows:

(8) $$\rho_{i\text{-}\mathrm{top}}=\frac{Z_{i}-Z_{i\sum }}{Z_{i}+Z_{i\sum }}$$

where $Z_{i\sum }$ is the equivalent impedance of each conductor, except conductor *i* in parallel.

The current reflection coefficient at the tower bottom can be expressed as follows:

(9) $$\rho_{t\text{-}g}=\frac{Z_{t}-Z_{g}}{Z_{t}+Z_{g}}$$

#### 3.1.2. Influence of Towers on the Lightning Channel Current

In the case of matching the characteristic impedance of different conductors and no current traveling wave reflection, it is distributed in $z^{\prime }>z$. The effect of current elements distributed in the lightning channel of $di_{1}(z,z^{\prime },t)$ on the current at observation point *z* can be expressed as follows:

(10) $$di_{1}(z,z^{\prime },t)=e^{-(z^{\prime }-h)/\lambda }f(t-\frac{z^{\prime }-h}{v}-\frac{z^{\prime }-z}{c})dz^{\prime }$$

In the case of considering the lightning traveling wave reflection at the connection point of each conductor and no back-flashover, the Formula (7) can be expressed as follows:

(11) $$\begin{array}{ll} di_{1}(z,z^{\prime },t) & =e^{-(z^{\prime }-h)/\lambda }dz^{\prime }\{f(t-\frac{z^{\prime }-h}{v}-\frac{z^{\prime }-z}{c})\\ \; & \;+\rho_{\mathrm{ch}\text{-}\mathrm{top}}f\left(t-\frac{z^{\prime }-h}{v}-\frac{z^{\prime }-z}{c}-\frac{2\left(z-h\right)}{c}\right)\\ \; & \;+\beta_{\mathrm{ch}\text{-}t}\beta_{t\text{-}\mathrm{ch}}\sum_{n=1}^{\infty }\rho_{t\text{-}g}^{n}\rho_{t\text{-}\mathrm{top}}^{n-1}\cdot f\left(t-\frac{z^{\prime }-h}{v}-\frac{z^{\prime }-z}{c}-\frac{2z}{c}-\frac{2\left(n-1\right)h}{c}\right)\} \end{array}$$

where *β_i_*_−*j*_ is the shunt coefficient transmitted from conductor *i* to conductor *j*, and the value of the shunt coefficient of different propagation paths can be determined from the equivalent circuit diagram after lightning strikes the tower; *n* is the number of reflections of lightning traveling waves on the tower top and the ground.

Due to the reflection of the current, the effect of the current element distributed in the lightning channel of $h<z^{\prime }<z$ on the current $di_{2}(z,z^{\prime },t)$ at observation point *z* can be expressed as follows:

(12) $$\begin{array}{ll} di_{2}(z,z^{\prime },t) & =e^{-(z^{\prime }-h)/\lambda }dz^{\prime }\{\rho_{\mathrm{ch}\text{-}\mathrm{top}}f\left(t-\frac{z^{\prime }-h}{v}-\frac{z^{\prime }-z}{c}-\frac{2\left(z-h\right)}{c}\right)\\ \; & \;+\beta_{\mathrm{ch}\text{-}t}\beta_{t\text{-}\mathrm{ch}}\sum_{n=1}^{\infty }\rho_{t\text{-}g}^{n}\rho_{t\text{-}\mathrm{top}}^{n-1}\cdot f\left(t-\frac{z^{\prime }-h}{v}-\frac{z^{\prime }-z}{c}-\frac{2z}{c}-\frac{2\left(n-1\right)h}{c}\right)\} \end{array}$$

At this time, the lightning current at the observation point *z* in the channel is jointly determined by Formulas (11) and (12), which can be expressed as follows:

(13) $$i(z,t)=\int_{z}^{H}di_{1}(z,z^{\prime },t)+\int_{h}^{z}di_{2}(z,z^{\prime },t)$$

Combining Formulas (4) and (6), the ideal current of the lightning current in which the return-stroke channel starts from the top of the transmission tower can be expressed as follows:

(14) $$i_{0}(h,t-\frac{z-h}{v})e^{-\left(z-h\right)/\lambda }=\int_{z}^{H}f(t-\frac{z^{\prime }-h}{v}-\frac{z^{\prime }-z}{c})e^{-\left(z^{\prime }-h\right)/\lambda }dz^{\prime }$$

According to Formulas (13) and (14), the distribution of the lightning current in the return-stroke channel originating from the tower can be obtained as follows:

(15) $$\begin{array}{ll} i(z,t) & =e^{-(z-h)/\lambda }i_{0}(h,t-\frac{z-h}{v})+\rho_{\mathrm{ch}\text{-}\mathrm{top}}i_{0}(h,t-\frac{z-h}{c})\\ \; & \;+\beta_{\mathrm{ch}\text{-}t}\beta_{t\text{-}\mathrm{ch}}\sum_{n=1}^{\infty }\rho_{t\text{-}g}^{n}\rho_{t\text{-}\mathrm{top}}^{n-1}i_{0}(h,t-\frac{z-h}{c}-\frac{2nh}{c}) \end{array}$$

Upon transmission tower back-flashover, due to the flashover of the insulator, the transmission line plays a shunting role. The wave impedance of the conductor should be taken into account for other factors, such as conductor coupling. After lightning strikes the tower, since the voltage amplitude of the lightning voltage acting on both ends of the insulator is uncertain, the moment to reach the critical breakdown voltage and the required discharge time delay cannot be determined. Breakdown will occur when the impulse voltage continues to increase, and the breakdown point may occur from the vicinity of the wave crest to the wave tail. Therefore, the analysis in this part also considers the occurrence of the back-flashover effect of time on the current distribution.

Based on the above considerations, it is assumed that the time when the lightning traveling wave reaches the tower top is the zero moment of back-flashover analysis. The insulator flashover occurs after time τ from the zero moment. When $m\cdot \frac{2h}{c}<\tau <(m+1)\cdot \frac{2h}{c},m\in N$, the current distribution of the lightning channel after the back-flashover can be expressed as follows:

(16) $$\begin{array}{ll} i(z,t) & =e^{-(z-h)/\lambda }i_{0}(h,t-\frac{z-h}{v})+\rho_{\mathrm{ch}\text{-}\mathrm{top}}i_{0}(h,t-\frac{z-h}{c})\\ \; & \;+N\beta_{\mathrm{ch}\text{-}t}\beta_{t\text{-}\mathrm{ch}}\sum_{n=1}^{m}\rho_{t\text{-}g}^{n}\rho_{t\text{-}\mathrm{top}}^{n-1}i_{0}(h,t-\frac{z-h}{c}-\frac{2nh}{c})\\ \; & \;+\beta_{\mathrm{ch}\text{-}t}{\bar{\beta }}_{t\text{-}\mathrm{ch}}\sum_{n=m+1}^{\infty }\rho_{t\text{-}g}^{n}\rho_{t\text{-}\mathrm{top}}^{m}{\bar{\rho }}_{t\text{-}\mathrm{top}}^{n-m-1}i_{0}(h,t-\frac{z-h}{c}-\frac{2nh}{c}) \end{array}$$

where ${\bar{\beta }}_{i\text{-}j}$ is the shunt coefficient of the lightning traveling wave transmitted from conductor *i* to conductor *j* after back-flashover occurs, ${\bar{\rho }}_{i\text{-}\mathrm{top}}$ is the reflection coefficient of lightning traveling wave passing through any conductor *i* connected to the tower top to reach the tower top being struck after back-flashover occurs, and *N* is the adjustment coefficient; when *m* = 0, *N* = 0, and when *m* > 0, *N* = 1.

#### 3.1.3. Distribution of Tower Lightning Current

When the lightning current observation point *z* is on the tower and no back-flashover occurs, the analysis method in the previous section can be used to obtain the effect of the current element at *z*′ on the observed current at point *z* as follows:

(17) $$\begin{array}{ll} di(z,z^{\prime },t) & =\beta_{\mathrm{ch}\text{-}t}e^{-(z^{\prime }-h)/\lambda }dz^{\prime }\{\sum_{n=0}^{\infty }[\rho_{t\text{-}g}^{n}\rho_{t\text{-}\mathrm{top}}^{n}f(t-\frac{z^{\prime }-h}{v}-\frac{z^{\prime }-z}{c}-\frac{2nh}{c})\\ \; & \;+\rho_{t\text{-}g}^{n+1}\rho_{t\text{-}\mathrm{top}}^{n}f\left(t-\frac{z^{\prime }-h}{v}-\frac{z^{\prime }-z}{c}-\frac{2z}{c}-\frac{2nh}{c}\right)]\} \end{array}$$

The comprehensive cumulative lightning current distribution at point *z* (0 < *z* < *h*) on the tower is as follows:

(18) $$i(z,t)=\int_{h}^{H}di(z,z^{\prime },t)$$

Furthermore, the relationship between the ideal current at the tower top and the lightning current distributed in the lightning channel can be expressed as follows:

(19) $$i_{0}(h,t)=\int_{h}^{H}f(t-\frac{z^{\prime }-h}{v}-\frac{z^{\prime }-h}{c})e^{-\left(z^{\prime }-h\right)/\lambda }dz^{\prime }$$

At this time, the lightning current distribution on the transmission tower can be expressed as follows:

(20) $$i(z,t)=\beta_{\mathrm{ch}\text{-}t}\sum_{n=0}^{\infty }[\rho_{t\text{-}g}^{n}\rho_{t\text{-}\mathrm{top}}^{n}i_{0}(h,t-\frac{h-z}{c}-\frac{2nh}{c})+\rho_{t\text{-}g}^{n+1}\rho_{t\text{-}\mathrm{top}}^{n}i_{0}(h,t-\frac{h+z}{c}-\frac{2nh}{c})]$$

### 3.2. Long-Distance Measurement of Lightning Current on the Tower Based on the POCT

In the long-distance lightning current measurement of the power transmission system, it is usually realized by measuring the lightning traveling wave propagating along the lightning protection line or transmission line. Due to the propagation through a long distance, the measurement result will be affected by the structure of the conductor itself. Therefore, the purpose of monitoring is mainly to take advantage of its wide monitoring range to locate lightning faults within a certain distance or capture key lightning parameter characteristics. In view of this application scenario, current projects often analyze the impact of tower shunt under lightning strikes and take the shunt coefficient as a constant for analysis. When an insulator flashover fault occurs, the lightning current discharge channels of lightning strike types, such as lightning strike towers and shielding strike lines, are different, and the measurement range of monitoring different conductors will also be affected differently.

In the study of the overvoltage in transmission lines, there are various simulation models for transmission line towers, including concentrated inductance models, single-wave impedance models, and multi-wave impedance models. The concentrated inductance model equates the tower to an inductance, ignoring the propagation process of traveling waves on the tower. The single wave impedance model treats the tower as a uniform wave impedance, which is more accurate when the tower height is low. Although the single-wave impedance model is superior to the concentrated inductance model, treating the tower as such a simple structure is too simplistic and not suitable for practical towers with complex structures and high heights. Referring to the practices of Yamada [29] and Ishii [30], this article divides the tower into four parts based on the upper, middle, and lower cross arms. The multi-wave impedance model not only considers the wave propagation on the tower, but also considers the change of the ground capacitance of different heights of the tower, so the result is more in line with the actual wave impedance of the tower.

It can be seen from the characteristics of wave impedance that the wave impedance of a vertical cylinder depends on the equivalent radius *r_Tk_* and height *h_k_* of the *k* th part of the cylinder. The wave impedance of a single vertical conductor can be described by the following empirical formula:

(21) ZTk=60(ln232hkrTk−2)

The wave impedance of the cross-arm part of the tower:

(22) $$Z_{Ak}=60\ln\frac{2h_{k}}{r_{Ak}}$$

where *r_Ak_* is the equivalent radius of the pole, and the tower cross arm is 1/4 the length of the cross arm.

In the case of support and no support, the actual measurement shows that the wave impedance of the multi-conductor system is reduced by about 10% after adding the support [31]. The wave impedance of each part of the support is *Z_Lk_*, and the length of the support part is 1.5 times that of the corresponding part of the main support.

**Figure 7:**
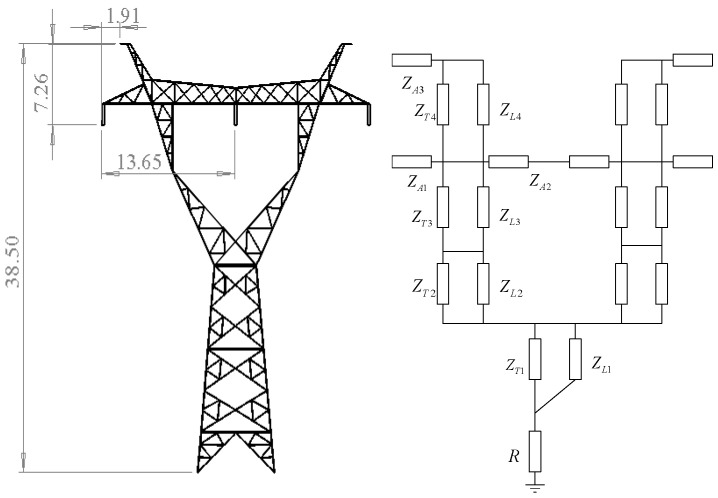
Tower diagram and equivalent model.

Taking the 220 kV transmission line wine cup type ZB_IV_ tangent tower as an example, the multi-wave impedance model is established. The schematic diagram and equivalent model of the tower are shown in Figure 7. The vertical body of the tower can be divided into four sections, and each cross arm is a section. The tower grounding resistance is replaced by ordinary resistance, and the grounding resistance is taken as 15 Ω.

When lightning strikes a transmission line, lightning rod, or ground wire, the overvoltage generated by lightning strikes will change the potential of the wire or tower insulator string. When the potential difference between the two ends of the insulator string is greater than the shock tolerance voltage, flashover accidents may occur. In our country, the line lightning protection design usually uses the flashover voltage of an insulator string with 50% flashover probability. The calculation is relatively simple, but the flashover characteristics in the breakdown process of the insulator are not taken into account. In the simulation analysis, whether the voltage waveform at both ends of the insulator or the horizontal extension of the maximum value crosses the volt-second characteristic curve of the insulator itself is taken as the criterion for whether flashover occurs. The volt-second characteristic calculation method of the insulator string can be calculated as follows:

(23) $$u\left(t\right)=400\cdot L_{x}+\frac{710\cdot L_{x}}{t^{0.75}}$$

where *t* is the time elapsed from lightning strike to flashover (μs).

The lightning withstand level of transmission lines is the main index to evaluate the reliable operation of transmission lines. The lightning withstand level of transmission lines in the simulation model built in this paper is 105 kA for counterattack and 12.8 kA for bypass. According to the provisions of industry standards [32], the lightning withstand level of the simulation model can meet the requirements of the regulations in the actual line and is equivalent to the specified value.

### 3.3. Measurement and Analysis of Key Lightning Current Parameters for Flashover Fault

**Figure 8:**
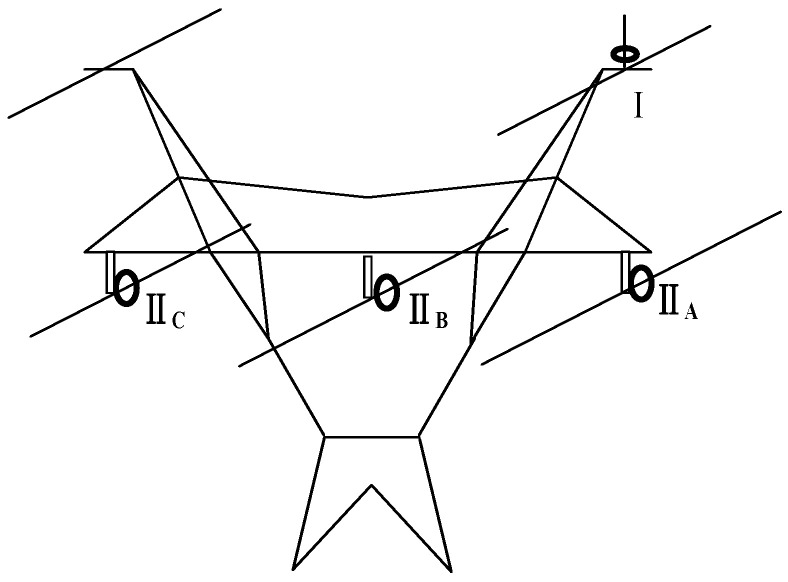
The design structure of the lightning current measuring method based on the POCT.

Considering the above analysis, the measurement structure is constructed based on the POCT, as shown in Figure 8.

This structure composed of two kinds of sensor units is called the POCT. One is set on the lightning rod on the tower top in the form of the direct measurement of the lightning current, which is used to measure the full waveform of the direct lightning strike. This paper calls it the waveform sensor unit, and its measurement position is called the tower top measurement position, which is represented by “I” in the diagram and the formula corner mark. The other is set on the three-phase line in the form of a remote measurement, which is used to measure the key parameters of the lightning current. It is called the parameter-sensing unit, and its measurement position is called the line measurement position. It is represented by “II” in the diagram and the formula corner mark, and the parameter- sensing unit on the I-phase of the line is represented by II*_i_* (*i* = A, B, C).

It can be seen from the analysis of typical lightning current parameters in the second part that most of the key information of lightning flow parameters, such as the amplitude, polarity, and rise rate of lightning flow, are concentrated on the rising edge of the lightning flow waveform. When different types of lightning strikes occur in the transmission system, the lightning current flows through different channels. Especially when insulator flashover fault occurs, the lightning current flows through the channel will change during the lightning strike, and the corresponding measuring channel along the rising edge of the lightning current will also change. Therefore, many scholars have measured the insulator flashover current.

#### 3.3.1. Distribution Characteristics of Lightning Current on the Tower Top

**Figure 9:**
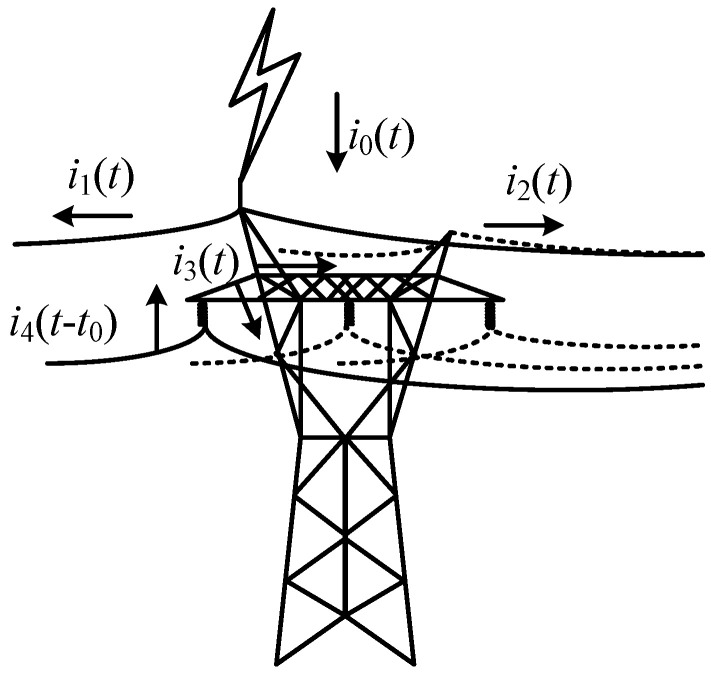
Current shunt diagram of lightning tower top.

The current shunt diagram of the lightning tower top is shown in Figure 9. *i*_0_(*t*) is the lightning current injected into the tower through the lightning protection line. The lightning current generates the current travelling wave *i*_1_(*t*) and *i*_2_(*t*) propagating to both sides of the lightning protection line. The direction of the lightning current flowing to the ground and the other side of the lightning protection line through the tower top is denoted as *i*_3_(*t*). Suppose the insulator flashover occurs at the time passing *t*_0_ after lightning strikes, the flashover current on the insulator is denoted as *i*_4_(*t* − *t*_0_), and the transient current of type I monitoring points on the tower top and type II monitoring points on the three-phase line are denoted as *i*_I_(*t*) and *i*_II*n*_(*t*) (n = A, B, C), respectively.

When no back-flashover occurs, each current component satisfies the following relation:

(24) $$i_{1}(t)+i_{2}(t)+i_{3}(t)=i_{0}(t)$$

Compared with the tower, the shunt system of the lightning protection line is small, so the amplitude of *i*_1_(*t*) and *i*_2_(*t*) is small. Even if the shunt coefficient is known to measure and restore *i*_0_(*t*) through *i*_1_(*t*) and *i*_2_(*t*), the signal-to-noise ratio will be low because of the small response monitored here, and it is difficult to measure the rising edge of the lightning current. However, the value will further decrease after several bases, thus increasing the difficulty of the measurement. *i*_I_(*t*) contains more original information than *i*_3_(*t*) before diverting several channels. Taking the fault phase A as an example, when the lightning tower flashover occurs, a transient current *i*_4_(*t* − *t*_0_) can be generated on the insulator, but since *i*_4_(*t* − *t*_0_) starts to act at time t_0_, there is no rising edge of the lightning current waveform. At this time, the A-phase line is connected to the tower through the broken insulator, and the traveling wave *i*_IIA_(*t*) of the transient current in the response will also be generated.

Due to the complex structure of the tower itself, through the tower will be connected with the lightning protection line, the ground, and back-flashover with the transmission line and the adjacent tower. Therefore, the current component measurement response *u_j_*(*t*) (*j* = 1, 2, 3, 4, I, II) of each lightning current channel starting from the response of the channel can be expressed as follows:

(25) $$u_{j}(t)=u_{j}^{\prime }(t)+u_{j}^{\prime \prime }(t)$$

where *u*′*_j_*(*t*) is the lightning current source in each channel current response; *u*″*_j_*(*t*) is the superposition of the response at time t of the refraction generated by the propagation of each lightning current channel to the wave impedance discontinuity.

#### 3.3.2. Distribution Characteristics of the Lightning Current on the Transmission Line

**Figure 10:**
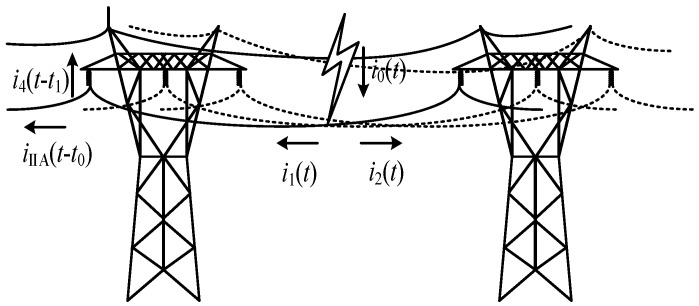
Current shunt diagram of shielding failure lines.

The current shunting diagram of shielding failure lines is shown in Figure 10. *i*_0_(*t*) is the lightning current injected into the transmission line, and the inflow time is zero. The lightning current generates the current traveling waves *i*_1_(*t*) and *i*_2_(*t*) propagating to both sides on the transmission line. It is assumed that the time passed by *t*_0_ after lightning strikes is transmitted to the tower where the monitoring system is located. The analysis in this section sets the lightning strike point on the line within a base tower and ignores the line attenuation within this distance. Suppose that the insulator flashover occurs at the time passing *t*_1_ after lightning strikes (*t*_1_ > *t*_0_) and the flashover current on the insulator is denoted as *t*_3_(*t* − *t*_1_).

In the case that the insulator flashover is not caused by shielding failure, each current component satisfies the following relation:

(26) $$i_{1}(t)+i_{2}(t)=i_{\mathrm{IIA}}(t-t_{0})+i_{2}(t)=i_{0}(t)$$

When the lightning current traveling wave propagates to the tower where the monitoring system is located and the insulator is not broken down, there is no insulator path shunt leading to the introduction of large *u_j_*″(*t*). The transmission line should contain relatively complete transient current information with less interference.

When lightning strikes the transmission line and causes flashover, each current component satisfies the following relation:

(27) $$i_{2}(t)+i_{\mathrm{IIA}}(t-t_{0})+i_{3}(t-t_{1})=i_{0}(t)$$

At this time, the insulator flashover connects the transmission line with the tower and shunt *i*_0_(*t*). It is assumed that the waveform peak of the lightning current component *i*_1_(*t*) at *t*_2_ reaches the insulator of the monitoring tower. When *t*_1_ < *t*_2_, insulator flashover occurs before the wave peak, and *i*_IIA_(*t*) includes the rising edge of *t*_0_~*t*_1_ in the lightning current. When *t*_1_ ≥ *t*_2_, *i*_IIA_(*t*) can contain the complete rising edge information, including the peak of the lightning current.

## 4. Simulation Analysis

### 4.1. Simulation Analysis of the Direct Measurement

This section is based on the POCT and determines the optimal measurement form of the sensing unit for the waveform measurement by studying the distribution characteristics of current traveling waves within the tower.

#### 4.1.1. Parameter Measurement Analysis

Cloud-to-ground lightning is the most common type of lightning, accounting for 90% of ground-to-ground lightning. About 80% of the negative cloud-to-ground lightning strikes contained two or more return strikes, and each lightning strike contained 3–5 return strikes on average. Therefore, the main form of a cloud-to-ground blitz is the first return stroke and the subsequent return stroke. These two are different in terms of the rise time and amplitude distribution. Aiming at these two most common lightning strike situations, the distribution model of the lightning current in the tower is used to analyze the distribution of the lightning current at the monitoring positions of the tower top and tower bottom in different heights of transmission towers.

**Figure 11:**
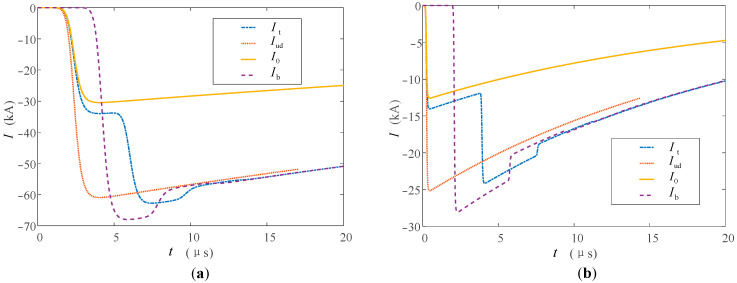
Measuring current at the top and at the foot of the high tower when (**a**) the first and (**b**) the subsequent return stroke occurs.

**Figure 12:**
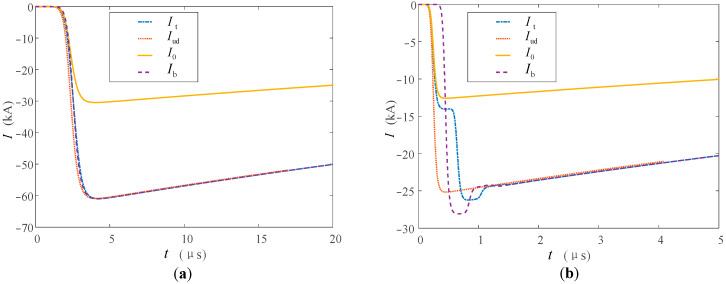
Measuring current at the top and at the foot of the low tower when (**a**) the first and (**b**) the subsequent return stroke occurs.

Refer to the typical data of Berger’s measurement of the first return stroke and the subsequent return stroke through the high tower recommended by CIGRE [33,34], and set the first return stroke and subsequent return stroke lightning current at the bottom of the lightning channel to be generated by the Heidler model [35]. The current peak value of the first return stroke is 30 kA, and the waveform is 2.4/78 μs; the current peak value of the subsequent return stroke is 12 kA, and the waveform is 0.25/20 μs. The tower heights of 553 m and 50 m are studied respectively. The above tower height selection refers to the Toronto CN tower [36] commonly used in lightning research and the straight-line tower height commonly used in 220 kV transmission lines to analyze the influence of tower height on the measurement results. In the simulation, the nonlinear process of the equivalent wave impedance of each part is ignored, and *β*_ch-t_ = 1.116, *ρ*_t-top_ = −0.115, *ρ*_t-g_ = 1. The simulation results are shown in Figure 11 and Figure 12.

It can be seen from Figure 11 and Figure 12 that the measurement results *I*_t_ and *I*_b_ at the top and bottom of the tower are all affected by the multiple refractions and reflections of the current traveling wave in the tower.

In the measurement of the high tower, *I*_t_ and *I*_b_ of the first return stroke are 31 and 68 kA respectively, and they are 14 and 28 kA in the subsequent return stroke. Before the top measurement point, the measurement results of the peak at the tower top are less affected. This is also because the traveling wave propagation time caused by the height of the tower is longer, so whether in the first return strike or in the subsequent return strike, the arrival time of the peak value of the lightning current at the tower top is closer to the original current waveform at the bottom of the channel. The difference between the peak arrival times of the two measurement points is about 2 μs.

When the lightning current return stroke is measured on the 50 m tower, the waveform characteristics of *I*_t_ and *I*_b_ are similar to those measured in the tall tower, but the peak arrival time difference is reduced to about 0.2 μs. This is due to the lower height of the tower, which further reduces the time for the lightning traveling wave to propagate in the tower. When the first return strike occurs, due to the double influence of the traveling wave head and the height of the tower, the amplitude measurement advantage at the tower top and the delay measurement error at the bottom of the tower are weakened, and the measurement results at the two positions are very close at this time.

Some scholars use the Norton equivalent circuit to analyze lightning strikes on high towers [37]. The current source in the equivalent circuit adopts the “interference-free current *I*_ud_” measured under ideal conditions, that is, the measured current when the tower height is ignored and *Z_ch_* ≫ *Z_g_*. Bringing the above conditions into Formula (20), we can obtain *i*(0,*t*) = 2*i*_0_(0,*t*), that is, *I*_ud_ = 2*I*_0_. Compared with *I*_0_, *I*_ud_ only changes proportionally in amplitude, so *I*_ud_ is added in the figure to compare the fluctuations of waveforms at different monitoring points. It can be seen from Figure 11 and Figure 12 that the waveforms of *I*_t_ and *I*_b_ are similar when the lightning current back-flashover is at the 50 m tower for the first time, and the waveforms measured in other cases are affected by the reflection of the tower. The waveform fluctuates the most when it reaches the peak, and the fluctuation gradually decreases and tends to be stable as time goes by, and the fluctuation of the measured value at the bottom of the tower is relatively large. Taking the first return stroke measurement in a high tower as an example, the maximum fluctuations of the waveform measurement values at the top and bottom of the tower are 3 and 8 kA, respectively.

Based on the above analysis results, when the tower height is low and only the first return strike is measured, the results of the two measurement positions are similar, and the tower top measurement results under other lightning strike conditions are better.

#### 4.1.2. Feasibility Analysis of Direct Measurements of the Lightning Current of Transmission Tower

**Figure 13:**
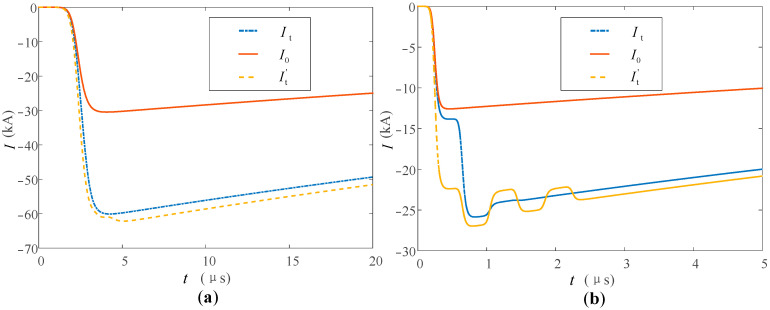
Comparison of the measurement current at the top of (**a**) the first return and (**b**) the subsequent return.

Simulate lightning strikes on transmission towers and ordinary towers. The measurement waveforms *I*_t_ and *I*_t_′ at the tower top are shown in Figure 13. Peak measurements were 14 and 22 kA in subsequent return strokes, respectively. This is due to the shunting effect of the lightning protection line of the transmission tower, which makes the refraction and shunt coefficient of the lightning current entering the tower lower. In addition, *I*_t_ has a longer time in the first return wave head, the waveform fluctuation is smaller, and the value is closer to the original lightning current amplitude after the larger fluctuation ends. Therefore, when the lightning current is measured on the transmission tower, the result obtained is closer to the current characteristics at the bottom of the channel, and the original lightning current information collection function can be realized.

### 4.2. Simulation Analysis of Remote Measurement

This section starts from the perspective of remote measurement of lightning current in the transmission system.

#### 4.2.1. Simulation Description

In the analysis, the lightning current source uses the Heilder source to simulate the 2.6/50 μs negative polarity lightning current. The lightning currents with amplitudes of 50 and 110 kA are respectively used to simulate the non-flashover and flashover conditions of lightning strikes on towers. The lightning current with the amplitude of 10 kA and 20 kA is taken respectively to analyze the situation of non-flashover and flashover of the shielding line.

**Figure 14:**
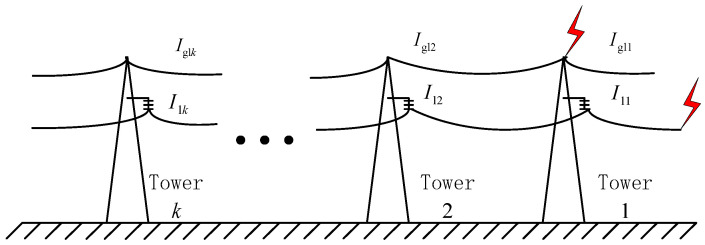
Schematic diagram of the lightning strike transmission line.

The schematic diagram of the transmission system in the analysis is shown in Figure 14. There are 10 base towers for the transmission system. In the simulation, the lightning strike point of the tower and the lightning strike line are located on the first base tower. The end of the line is simulated into a wireless long line by the setting. Set the absolute value of the initial lightning current amplitude to *I*_0_. The setting back-flashover was generated by the lightning strike on the tower. The fault phase and the strike phase of the back-flashover occurred on the same side A, and the flashover phase and the observed lightning protection line were on the same side. After the lightning current strikes with different amplitudes were investigated, the peak value of the lightning protection line current is measured on the *k* tower *I*_gl*k*_, the peak current of each phase *I*_l*k*_.

#### 4.2.2. Shielding Failure Analysis of the Transmission Line

When shielding failure occurs in the line, the absolute value distribution of the peak current at each base tower under the two conditions of non-flashover and flashover is shown in Table 1.

When shielding failure is non-flashover, the lightning current spreads along the transmission line to both sides, there is no obvious shunt channel, and the variation of the measured amplitude at each base tower is small. The lightning current on the lightning protection line is mainly coupled by the transmission line, and its amplitude is small. Since the amplitude of the lightning current around the striking phase changes little, the lightning current on the corresponding lightning protection line is basically unchanged.

**Figure 15:**
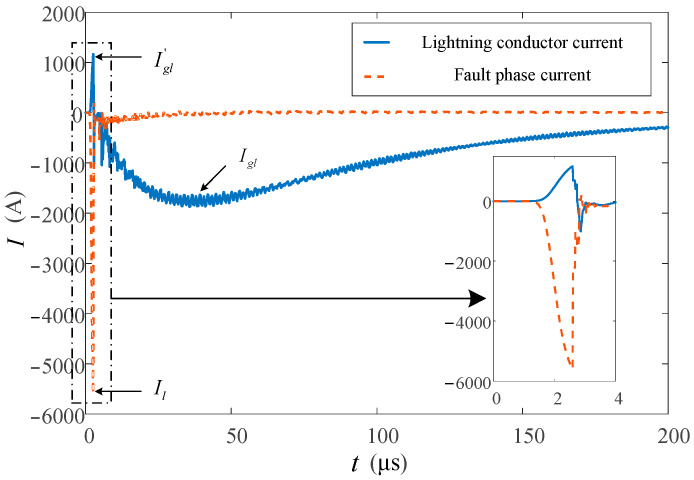
Typical waveforms of *I*_gl_, *I*_gl*k*_, and *I*′_gl*k*_ in the fault phase.

After the flashover, the lightning current in the transmission line will enter the ground through towers. Therefore, *I*_l2_ is going to drop off a little bit; due to the insulator flashover, part of the lightning current *I*′_gl*k*_ flows from the tower into the lightning protection line, so the difference between two polar values in the lightning protection line and tower is quite large. Taking the waveforms measured at the first base tower as an example, the typical waveforms of *I*_gl_, *I*_gl*k*_, and *I*′_gl*k*_ on the fault phase side (phase A) are shown in Figure 15. *I*_gl*k*_ is mainly induced by the struck phase, so it is consistent with the changing trend of the struck phase current, and the amplitude of *I*′_gl*k*_ decreases gradually with the increase in the number of passing towers.

**Figure 16:**
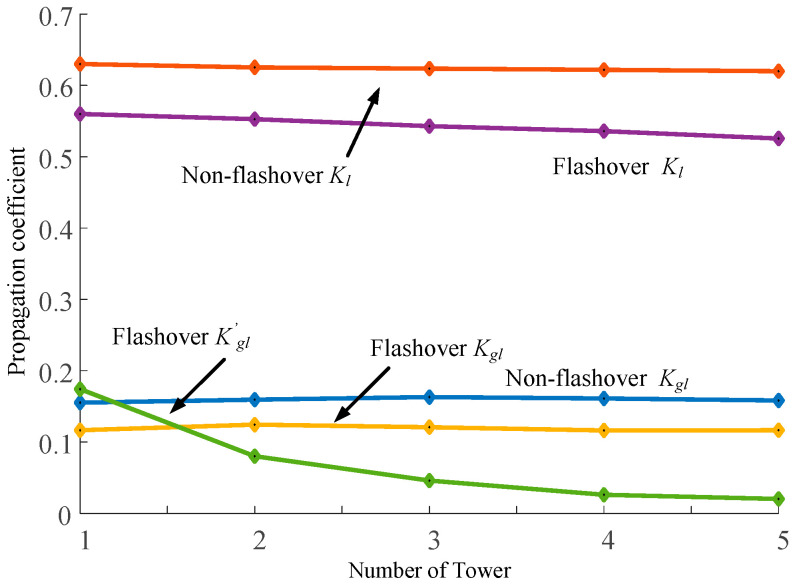
Propagation coefficient trend when lightning strikes the transmission line.

The propagation coefficients *K*_gl_ = 2*I*_gl*k*_/*I*_0_, *K*_l_ = 2*I*_l*k*_/*I*_0_, and *K*′_gl_ = 2*I*′_gl*k*_/*I*_0_ are introduced to evaluate the lightning current measurement range of the lightning protection line and transmission line, respectively. Figure 16 shows the variation in *K*_gl_, *K*_l_, and *K*′*_g_*_l_ of the aside lightning protection line with the maximum response and the number 1–5 towers of the A-phase conductor.

As can be seen from Figure 16, in the case of non-flashover, since lightning flow is concentrated in the transmission line, the initial value of *K*_l_ shows an overall downward trend from about 0.63, but the decline is gentle and the change is small. After passing through 5-tower, it can still stabilize at about 0.62. The coupling quantity of the lightning current propagating in the lightning protection line is small, the initial value of *K*′*_g_*_l_ is about 0.16, and the overall trend is consistent with the change in *K*_l_.

When flashover around occurs, the changing trend of *K*_l_ is basically unchanged, but the value decreases further than that without flashover. Compared with other cases, the starting point of *K*′*_g_*_l_ is about 0.18, but it will drop obviously after passing one base tower and decreases to 0 after 10-tower. The initial value of *K*_gl_ is about 0.12, which further decreases and changes little compared with that without flashover, and the overall trend is consistent with that of *K*_l_.

#### 4.2.3. Analysis of Lightning Strikes on Towers

When lightning strikes towers, the distribution of the absolute value of the peak current at each base tower under the two conditions of no back-flashover and back-flashover is shown in Table 2.

When there are no lightning back-flashovers at the tower top, the lightning current mainly enters the ground through the tower and is partially shunted by the lightning protection line. When the lightning current propagates along the lightning protection line and passes through the next tower, the lightning current is further shunted, and the amplitude of *I*_gl_ also decreases correspondingly. The lightning current in the transmission line is coupled from the lightning protection line, so the amplitude is small and the change is small after passing through the base tower.

**Figure 17:**
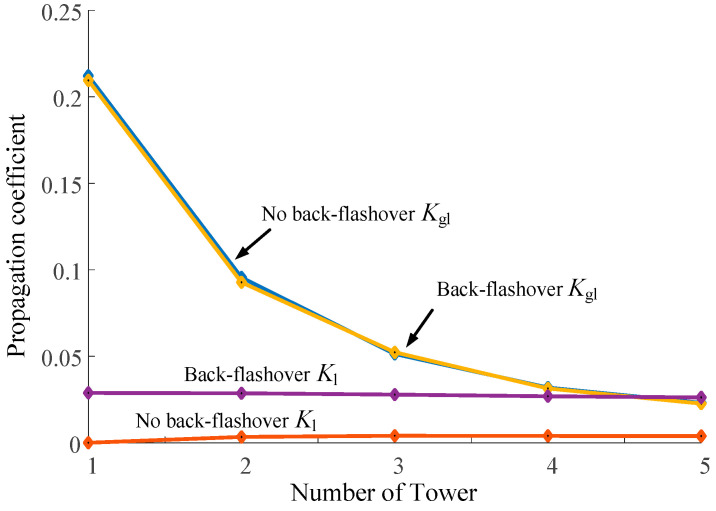
Propagation coefficient trend when lightning strikes the top of the transmission tower.

When lightning strikes the tower, the *I*_gl_ value on the lightning protection line will drop sharply from the first tower due to the shunt of the flashover phase, and then, the *I*_gl_ value after passing through each base tower will drop on this basis. Also affected by the shunt of the flashover insulator, the *I*_l_ value will increase. Since the propagation path is unchanged, the subsequent *I*_l_ value of the multi-base tower will change little. The changing relationship between *K*_gl_ and *K*_l_ is shown in Figure 17.

As can be seen from Figure 17, in the case of no back-flashover, the lightning current propagation of the lightning protection line will also be affected by the tower shunt. The initial value of *K*_gl_ is relatively large, about 0.21, and it will be significantly reduced after each tower passes through one base, about 0.02 after passing through five base towers. Because the lightning current of the transmission line is coupled, the initial value of *K*_l_ is small and the overall changing trend is small, about 0.004.

When back-flashover occurs, the propagation of the lightning current in the lightning protection line does not change, and the overall trend is basically the same as that without back-flashover. After the injection of the lightning current by the flashover insulator, the initial value of *K*_l_ is greatly increased, about 0.028. The overall trend is downward but relatively gentle. After five towers, the value is already greater than *K*_gl_.

From the above analysis, it can be seen that the initial value and the overall trend of the change i *K*_l_ are much larger than that of *K*_gl_ when the attack occurs. When strike back occurs, the initial value of *K*_gl_ is greater than *K*_l_, but there is little difference between the two values after four towers, and the value of *K*_l_ is greater than *K*_gl_ after five towers. This indicates that under different lightning strike conditions, the measured values of transmission lines in the measured distances of five towers or more are closer to the initial values of the lightning current. Therefore, setting the remote monitoring point on the transmission line can give full play to the advantage of the large-scale measurement and provide a research basis for the location of the lightning strike line and the measurement of key parameters.

### 4.3. Measurement and Analysis of Lightning Current Parameters for the Flashover Fault

From the point of view of lightning current key parameter measurements, this part analyzes the lightning current measurement response of the tower top, three-phase line, and insulator under different lightning strike types and studies the feasibility of the proposed measurement structure and the necessity of the insulator flashover current measurement.

#### 4.3.1. Lightning Strike on the Tower Top without Flashover

**Figure 18:**
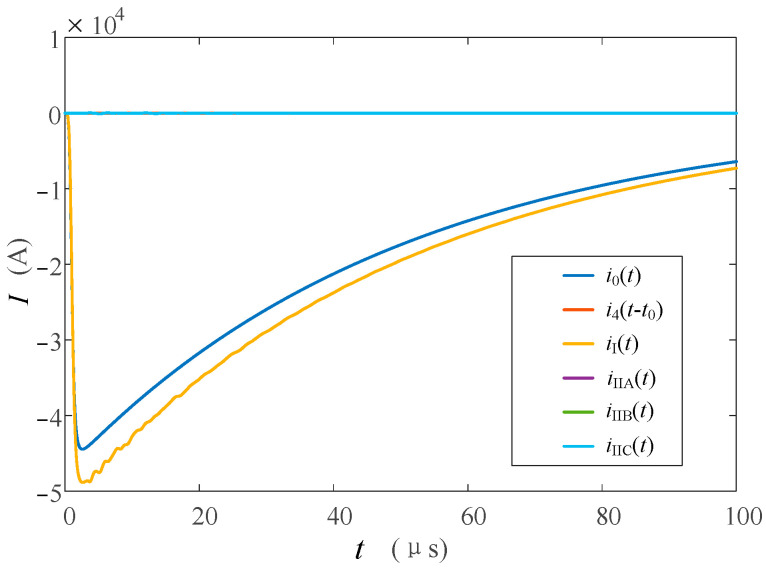
Current waveforms in each measuring position when lightning strikes the tower top without flashover.

**Figure 19:**
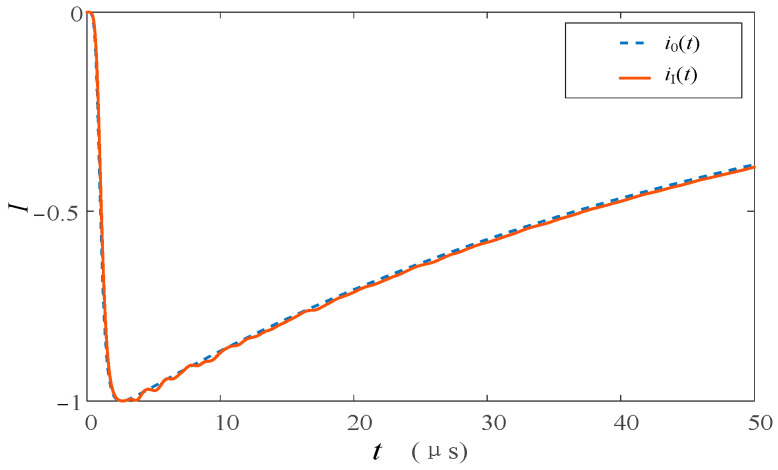
Comparison of current waveforms between *i*_I_(*t*) and *i*_0_(*t*) when lightning strikes the top of the transmission tower without flashover.

The simulation model is used to analyze the current traveling wave of each observation point. When lightning strikes and no flashover occurs at the tower top, each transient current waveform is shown in Figure 18. Since the tower and the transmission line do not form a pathway, there is no flashover current flowing through *i*_4_(*t* − *t*_0_) and no response on *i*_II*n*_(*t*) connected to it. Since *i*_I_(*t*) is before the shunt of the following channel, the response of *i*_I_(*t*) at each monitoring point is the maximum and the polarity is the same as that of lightning. Compared with *i*_0_(*t*), it can be seen that the wave amplitude of *i*_I_(*t*) is large and there is a certain oscillation due to the folding and reflection phenomenon between the tower and the ground, lightning protection line, and other wave impedance discontinuous conductors. *i*_I_(*t*) and *i*_0_(*t*) were normalized, and the waveform comparison is shown in Figure 19. As can be seen from Figure 19, the waveforms of *i*_I_(*t*) and *i*_I_(*t*) are very consistent, and the leading edge of the current wave is very close.

#### 4.3.2. Lightning Strikes on the Tower Top Flashover

**Figure 20:**
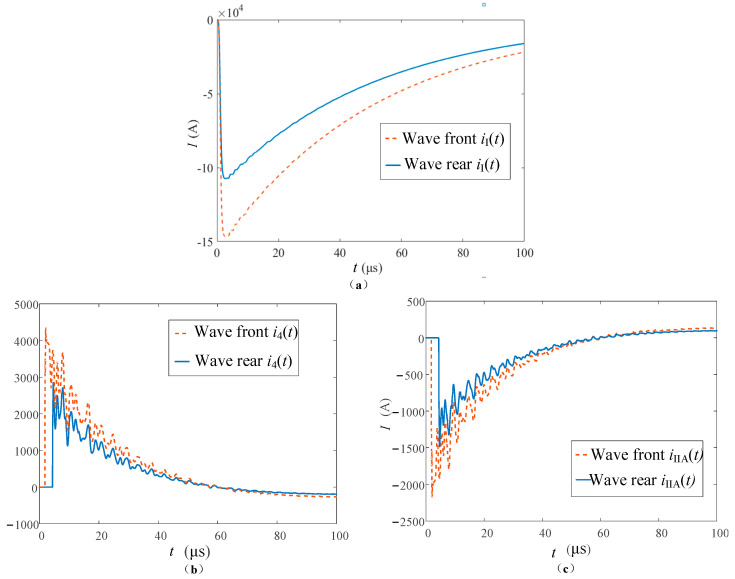
Current waveforms in each measuring position when lightning strikes the tower top with flashover; (**a**) tower top current, (**b**) insulator current, and (**c**) fault phase current.

When insulator flashover occurs, the flashover moment may occur at the rising edge before the lightning current peak or may occur after the peak. The lightning current of 150 and 110 kA is passed on the tower top respectively to simulate the wave front and wave rear flashover of A-phase insulators. The transient waveforms of each lightning current channel are shown in Figure 20.

The lightning current at the tower top is shown in Figure 20a. After the tower top is struck by lightning, there is a large lightning current flowing through the type I monitoring point as it is in front of the diverging channels. Therefore, *i*_I_(*t*) has a larger amplitude of 10.5 and 14.5 kA, and the polarity is the same as the lightning current. The insulator flashover current is shown in Figure 20b. Different amplitudes cause different moments of insulator flashover. Since the flashover time is near the wave peak, there is no process of rising amplitude after the flashover, and the attenuation starts from a relatively large value. Compared with the type I monitoring point, the waveforms of the two groups showed the same changing trend, and the responses were smaller, with the maximum peaks of about 4260 and 2850 A, respectively. In addition, due to the high peak value and relatively small response at the flashover time, the influence of *u_j_*″(*t*) in the formula is particularly serious and the amplitude of oscillation is large. The lightning current in the fault phase is shown in Figure 20c, because the lightning current measured at the type II monitoring point on fault phase A is transmitted to the line through the insulator. Therefore, the waveform characteristics and variation trend of *i*_IA_(*t*) are consistent with those of *i*_4_(*t* − *t*_0_) under two kinds of lightning current strikes. However, the overall response is smaller than that of *i*_4_(*t* − *t*_0_) due to the shunt effect.

**Figure 21:**
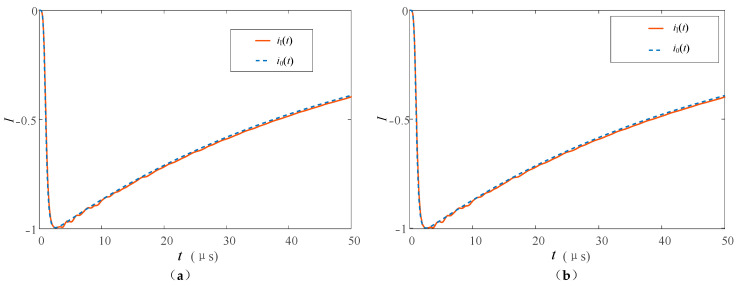
Comparison of current waveforms between *i*_I_(*t*) and *i*_0_(*t*) when lightning strikes the top of the transmission tower with flashover; (**a**) flashover occurs before the peak and (**b**) flashover occurs after the peak.

The result of the normalization of *i*_IA_(*t*) and *i*_0_(*t*) is shown in Figure 21. The *i*_I_(*t*) current in the two cases of the wave front and wave back flashover will have a slight oscillation under the influence of *u_j_*″(*t*). However, because there is less number of insulator flashover, there is little difference between the waveform of the lightning current and that of the top of the lightning tower without flashover, and the waveform front is in good consistency with *i*_0_(*t*).

#### 4.3.3. Lightning Strikes on the Transmission Line without Flashover

**Figure 22:**
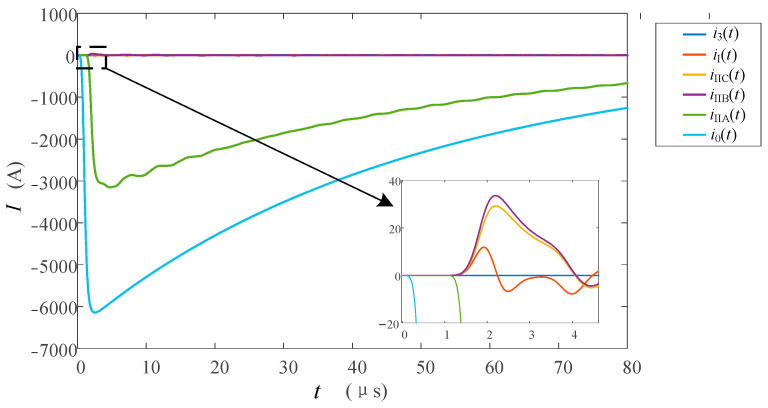
Current waveforms in each measuring position when lightning strikes the A-phase wire without flashover.

The simulation analysis of the lightning current strikes on the transmission line without flashover is carried out, and the transient current waveform flowing through each channel is shown in Figure 22. *i*_IIA_(*t*) has the maximum response, and the polarity is the same as that of the lightning current source because there is no tower shunt. There is no lightning current on the insulator. *i*_IIB_(*t*) and *i*_IIC_(*t*) are derived from the common coupling between the fault phase and the induced current on lightning protection line. Since the above coupling currents are negative, the polarity of *i*_IIB_(*t*) and *i*_IIC_(*t*) is positive.

In addition, *i*_IIB_(*t*) and *i*_IIC_(*t*) have a smaller amplitude and larger distortion than *i*_IIA_(*t*) and *i*_0_(*t*) due to the short propagation distance of the lightning current from the lightning strike point to the monitoring point and inconsistent arrival time of the amplitude of each coupling source. *i*_I_(*t*) will have a smaller amplitude and a larger oscillation response because of the smaller portion of the inductive current in the lightning line flowing into the tower.

**Figure 23:**
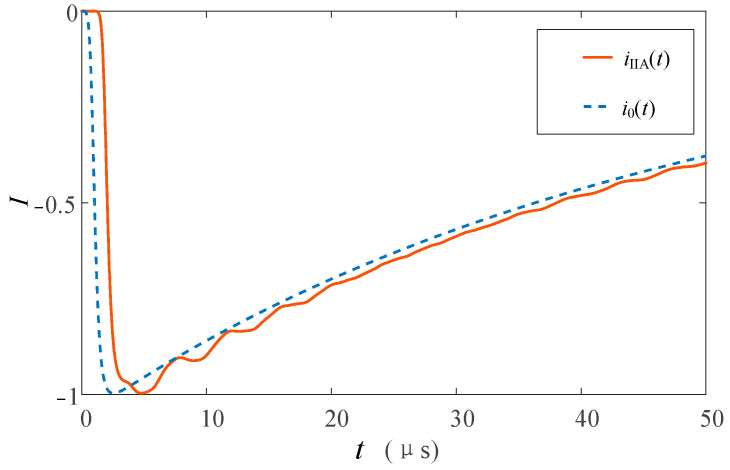
Comparison of current waveforms between *i*_IIA_(*t*) and *i*_0_(*t*) when lightning strikes the A-phase wire without flashover.

*i*_IIA_(*t*) and *i*_0_(*t*) are normalized, and the waveform comparison is shown in Figure 23. As can be seen from Figure 23, the *i*_IIA_(*t*) waveform will be affected by *u_j_*″(*t*) to produce a small amplitude fluctuation. However, it is very consistent with the waveform of *i*_0_(*t*); especially, the leading part of the current wave is very close.

#### 4.3.4. Lightning Strikes on the Transmission Line with Flashover

**Figure 24:**
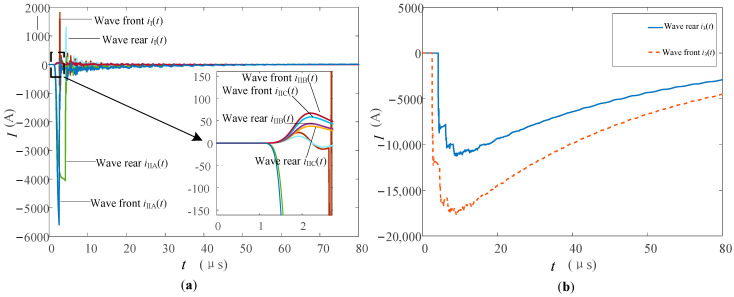
(**a**) Current waveforms in each measuring position, and (**b**) insulator flashover current waveforms when lightning strikes the A-phase wire with flashover.

When flashover of shielding failure occurs, the amplitude of the lightning current source is set as 20 and 13 kA, respectively, at the lightning strike point to simulate the flashover before and after the wave crest. The transient waveforms of each lightning channel are shown in Figure 24a. The flashover current waveforms of insulators under two flashover conditions are shown in Figure 24b.

In Figure 24a, it can be seen that, because the insulator flashover produces a large lightning current into the tower, the *i*_I_(*t*), compared to the detour lightning flashover response, increases in size by about two orders of magnitude. The starting time of the two waveforms in the diagram corresponds to the insulator flashover time respectively. In addition, due to the action of *u_j_*″(*t*), the waveform of *i*_I_ continues to oscillate until the attenuation reaches zero, so its waveform cannot reflect the rising edge of the lightning current.

As can be seen from Figure 24b, the amplitude of the lightning current acting on the insulator at the moment of the wave front and back flashover is large, so the amplitude at the moment of flashover is large. In addition, the *i*_3_(*t*) waveform presents the characteristics of stepping up and then oscillating down due to the lightning current connected to the tower at the moment of the flashover, and the changing trend of the waveforms is consistent.

*i*_3_(*t*) in both cases could not measure the rising edge. The peak could not be measured due to the action of *u_j_*″(*t*). Before insulator flashover, the waveform of *i*_IIA_(*t*) is similar to that of *i*_IIA_(*t*) when the flashover is not struck. When flashover occurs, most of the lightning current flows into the tower, and *i*_IIA_(*t*) falls instantly in both cases of flashover. The oscillation is attenuated by the action of *u_j_*″(*t*). The waveform characteristics of *i*_IIB_(*t*) and *i*_IIC_(*t*) in the two cases of flashover are consistent with those without flashover, and the response is small, which cannot reflect the rising edge information of the lightning current.

**Figure 25:**
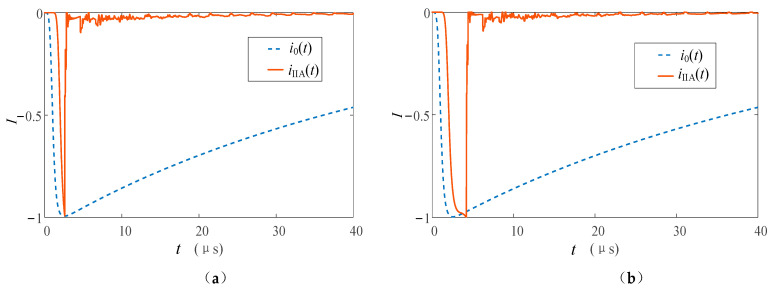
Comparison of current waveforms between *i*_IIA_(*t*) and *i*_0_(*t*) when lightning strikes the A-phase wire with flashover (**a**) flashover occurs before the peak (**b**) flashover occurs after the peak.

The results of normalization of *i*_IIA_(*t*) and *i*_0_(*t*) are shown in Figure 25. The *i*_IIA_(*t*) current under the two types of flashover conditions will have small oscillations under the influence of *u_j_*″(*t*). The waveform of the lightning current is similar than that of the circuit without flashover. *i*_IIA_(*t*) can measure part of the rising edge information of the lightning current upon wave front flashover. *i*_IIA_(*t*) can measure the rising edge information of the lightning current, including the peak value when the wave is in flashover behind the wave.

To summarize, when lightning strikes the tower top, whether it is flashover or not, the sensor unit of type I on the tower top can measure the original waveform of the lightning current well. Upon insulator flashover, the transient current will flow through the insulator and the fault phase connected to the flashover insulator. However, the initial time has already missed the rising edge of lightning current, and the amplitude is too small to measure the key parameters of the rising edge of the lightning current. When the insulator is not in flashover, the type II sensor of the fault phase can measure the waveform of the lightning current better. In the event of flashover, the type II sensor unit of the fault phase can measure the key parameters of the rising edge of the lightning current. From the perspective of measurements of lightning current key parameters, the lightning current measurement system of the transmission line proposed in this section can be used to measure lightning current key parameters under different lightning strikes without setting insulator flashover current measurement points.

## 5. Conclusions

The measurement results and efficiency will be affected by different conductors through which lightning current flows and different monitoring positions of the same conductor. In this paper, the lightning current measurement of the transmission system is taken as the background, and the sensor monitoring forms of the direct lightning current measurement of the transmission tower and the remote lightning current measurement of the line are analyzed.

(1) The current propagation model of the pole tower is established. The model describes the distribution of current elements in the tower and the influence of the transmission tower itself on the measurement results of the lightning current. The distribution of the lightning current in different positions of the tower after lightning strikes the pole tower is analyzed. The measurement position of the direct lightning strike at the tower top is determined from the angle of the accurate measurement of the original lightning current.
(2) Build a simulation model of a multistage tower transmission system, and analyze the transmission characteristics of the lightning current in transmission lines under different lightning strikes. The results show that the response of lightning current measurements in the transmission line is higher than that of lightning protection line, and it is more suitable for remote measurements. When lightning strikes a tower, the response of the lightning rod is large regardless of whether there is lightning back-flashover, but the attenuation is faster with an increase in the distance. After the distance of five base towers, the lightning current measurement of the lightning rod and tower is more suitable for the remote measurement.
(3) Construct the lightning current measurement structure of the transmission system. From the perspective of measuring the rising edge of the lightning current waveform containing more critical lightning current information, analyze the measurement results of the type I and II monitoring points and insulator flashover current measurement points under different lightning strikes. The analysis shows that the fault current of the insulator cannot measure the key parameters of the rising edge, the type I sensor unit can measure the rising edge of the lightning current waveform when lightning strikes the tower, the type I sensor unit cannot measure the lightning current waveform when lightning strikes and flashover occurs, and the type II sensor unit can measure part of the rising edge information of the lightning current upon wave front flashover. The type II sensor unit can measure the rising edge information of the lightning current, including the peak value when the wave flashover is behind.
(4) When lightning strikes towers or lines, regardless of whether there is insulator flashover or not, the response of the lightning current measurement on the transmission line is higher than that on the lightning protection line, which is more suitable for remote measurements. The insulator flashover current under different fault conditions is studied from the point of view of critical lightning current measurements. The results show that the insulator flashover current has limitations in the measurement of the key parameters of the lightning current. The direct measurement and the remote measurement can replace the insulator flashover current measurement.

## Figures and Tables

**Table 1:** Distribution of the lightning current when lightning strikes the transmission line.

Form of Lightning Strike	Number of Tower *k*	*I*_gl*k*_ (*I*′_gl*k*_) (A)		*I*_l*k*_ (A)		
		A-Phase Side	C-Phase Side	A-Phase	B-Phase	C-Phase
shielding failure without flashover	1	777	292	3150	33	29
	2	797	295	3127	65	55
	3	815	314	3118	97	82
	4	806	306	3109	126	106
	5	792	292	3100	154	128
shielding failure with flashover	1	1166 (1742)	27 (1703)	5600	67	58
	2	1243 (801)	375 (725)	5526	135	115
	3	1209 (460)	351 (423)	5428	195	165
	4	1163 (260)	314 (274)	5359	253	212
	5	1166 (203)	301 (195)	5257	308	256
	10	1120 (0)	360 (0)	4890	600	492

**Form of Lightning Strike**

**Number of Tower *k***

***I*_gl*k*_** **(*I*** **′** **_gl*k*_** **) (A)**

***I*_l*k*_ (A)**

**A-Phase Side**

**C-Phase Side**

**A-Phase**

**B-Phase**

**C-Phase**

**Table 2:** Distribution of the lightning current when lightning strikes the top of the transmission tower.

Form of Lightning Strike	Number of Tower *k*	*I_glk_* (A)		*I_lk_* (A)		
		A-Phase Side	C-Phase Side	A-Phase	B-Phase	C-Phase
lightning stroke the tower without back-flashover	1	5303	292	0	0	0
	2	2390	295	83	81	83
	3	1284	314	101	99	100
	4	797	306	100	98	100
	5	577	292	98	95	97
lightning stroke the tower with back-flashover	1	11,523	11,420	1587	0	0
	2	5105	5185	1576	173	182
	3	2876	2758	1534	218	223
	4	1729	1766	1480	220	225
	5	1244	1281	1444	213	217

## Data Availability

The research data are unavailable due to the project funding’s confidentiality.
